# City classification and health burden: Evidence from U5 malaria in the rapidly growing city of Akure, Nigeria

**DOI:** 10.1016/j.ijregi.2024.100515

**Published:** 2024-12-12

**Authors:** Taye Bayode, Olumuyiwa Bayo Akinbamijo, Alexander Siegmund

**Affiliations:** 1 Institute of Geography & Heidelberg Centre for Environment, Heidelberg University, Heidelberg, Germany; 2 Department of Geography – Research Group for Earth Observation (rgeo), UNESCO Chair on World Heritage and Biosphere Reserve Observation and Education, Heidelberg University of Education, Heidelberg, Germany; 3 Department of Urban and Regional Planning, School of Environmental Technology, Federal University of Technology Akure, Nigeria

**Keywords:** ANOVA, Cities, Informal settlement, Urban development, Nigeria

## Abstract

**Objectives:**

Little is known about the complex interplay between urban structure and health in rapidly urbanizing cities in Nigeria.

**Methods:**

The study broadly used very high-resolution satellite imagery and gathered primary data. With the aid of the very high-resolution imagery and identified neighborhoods, two neighborhoods each were sampled based on their classified urban structure characteristics. The obtained malaria epidemiologic data were aggregated according to stratified neighborhoods. Analysis of variance was used to determine the mean difference of malaria burden between the spatial structure.

**Results:**

The study shows that under 5 malaria varies significantly among the settlement classes (*P* = 1.118e-05) with highest difference between peri-urban settlement and medium-density settlement (*P* = 0.0000988). The study shows higher malaria burden in the informal and peri-urban settlement compared with the lower prevalence in formal settlements.

**Conclusions:**

The study findings underscore the urgent need for comprehensive urban development policies to address health inequities by prioritizing physical infrastructure improvements in marginalized areas. Such improvements will translate to enhanced health equity for rapidly growing informal, poor, and deprived settlement areas and ultimately promote sustainable urban development efforts.

## Highlights

• There is intra-urban variability of health outcome in Akure, Nigeria.
• Disease burden is high in the city center and newly emerging areas of Akure.
• Spatial structure has indirect effect on health outcome in Akure, Nigeria.

## Introduction

Cities play important and critical role in the achievement of Sustainable Development Goal 3. However, the urbanization trend in low- and middle-income countries, such as Nigeria, presents significant sustainable development challenge, particularly, the striking urban health inequalities. It is well-known that factors such as geography, socio-ecological characteristics, and health policies influence health inequality; little is known about the complex interplay between urban structure and health in rapidly urbanizing cities in Nigeria.

Urban structure, also known as urban spatial structure (USS), illustrates the structural patterns in cities and extended urban areas. It is of note that within a city, the spatial structure landscape can vary dramatically from the Central Business District to the suburbs. This is often as a result of dynamic economic structures with accompanied transformation in the city's socio-spatial organization [1]. Urban economists model USS based on the complex and interacting economic forces of decentralization, dispersion, and multiple employment [2]. This analogy is not far dissimilar from the tenets of the Chicago school's description of city growth and development patterns. A simpler illustration was inferred by Zhang et al. [3] as changing distribution of population in the urban space as a result of economic activities and average decline in urban density gradient within enlarged urban spatial limit [4]. Urban population and density, economic activities, level of education, health, and segregation are some of the key determinants of urban structure, thereby forming the theoretical conceptualization of neighborhoods [5,6]. This holds true in Akure, with diverse urban density structure. Classification pattern of Akure into residential structures dates to the work of Olorunfemi where housing in Akure were characterized due to the rapid development, population growth, and changing political status [7]. The rapid development and population growth of Akure is asserted to be a continuous phenomenon. According to the study by Urbanization Research Nigeria in 2015, Akure is identified as a city that will experience major concentration of urban spatial growth and physical development because Nigeria's urbanization rate was projected to be about 52% in the year 2020 [1].

**Figure 1:**
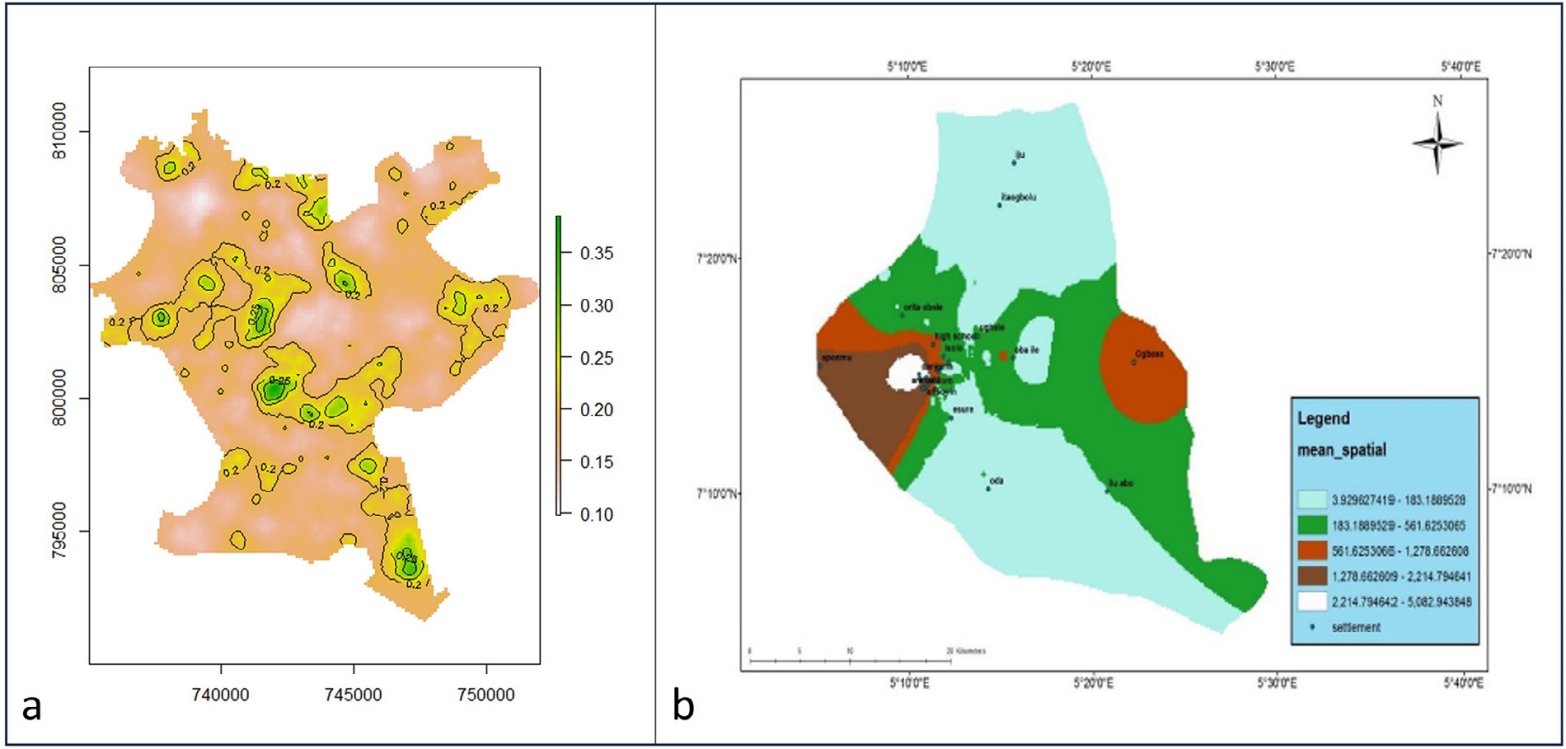
(a) Spatial pattern of under 5 malaria prevalence in Akure [11]. (b) Mean spatial distribution of malaria prevalence in Akure [12].

Furthermore, the complex interaction between population increases and skewed distribution of services exacerbates the inflow impact of population from rural hinterlands around Akure to the city of Akure. This has resulted in the emergence of new places in the suburbs. However, these newly emerged urban areas often experience shortages of services and infrastructure due to demographic demands and population density [8]. Ultimately, the new urban areas and/or urban expansion is likely to increase in Nigeria, with profound consequences for the physical configuration of Nigerian urban settlements and impacts on the health of urban residents [9,10]. For example, [11] identification of children below the age of 5 years (U5) malaria variability in Akure reveals the enormous burden in poor and low-income residential communities, i.e. city centers (Arakale, Araromi) and newly emerging areas (suburbs such as Oba Ile), as shown in Figure 1. Similarly, Abdulkareem et al. [12] observed the highest burden of malaria in core areas of Akure such as Araromi, Arisoyin, High School, and Ogbese, followed by relatively high burden in the suburbs such Orita Obele, Esure, and Oba Ile.

Scholars have investigated impacts of residential or spatial structure in Akure on various aspects. For example, Olujimi [13] explored how disparities in infrastructure between different zones in Akure impacts rental values of residential properties, and Emmanuel [14] explored socio-economic gradient along residential densities in Akure, whereas Olayiwola [15] explored the correlation between socio-economic characteristics and quality of residential neighborhoods in Akure. Akinbamijo and Fasakin [16] explored spatial disparities of health among residential zones (core, transition, and peripheral) in Akure. However, the study does not empirically classify these residential neighborhoods and did not investigate the impact (direct/indirect) on residents’ health. Earlier, Alex et al. [2] pointed out the need to study urban structure because city growth pattern is experiencing qualitative change. However, to date and to the best of the authors’ knowledge, no studies have explored the impact (direct or indirect) on health of Akure residents. Our study aims to fill this identified gap using childhood malaria prevalence as a proxy for health condition in Akure. To date, malaria remains a significant public health problem in Nigeria. Nigeria records the highest cases of malaria and deaths worldwide. Worse, the burden of malaria and death toll are higher among U5 [17,18].

### Urban land use model: a brief overview

Historically, USS widely spread model with more applicability in urban land use planning and distribution of population are the ecological models—concentric zone, sector, and multiple nuclei models. Other approach includes economic model and activity models as rightly put forward by Carter [19]. The concentric zone model was developed by Burgess [20,21] to explain urban social structures in cities as increasing socio-economic status with distance from the city center—a concept adopted from plant ecological framework (competition, dominance, invasion, and succession). Alternatively, Adams and Hoyt [22] described urban structure and city growth pattern based on residential rent pattern and impacts of transportation development with the sector model, a theory propounded by Hoyt, H. in 1939. Sector and concentric zone models overlap. Although both models explain the outward growth of cities, their practicality, structure of growth, and land use patterns differ. The former has practical application in real estate management with radial outward growth and non-random land use pattern, whereas the latter has a sociological application, with ring-like outward growth and random land use pattern. Often, large cities do not grow a single nucleus. Thus, formal and more effective generalization of urban land uses were developed by Harris and Ullman in 1945 [23]. The basic assumption is that city's mini nuclei was originally developed independently with the specialized advantages that they offered [6]. The delineation or classification of neighborhoods based on morphological characteristics is complex, multidimensional, and nebulous [5]. Nevertheless, the emergence of new data sources and availability of increased computational power has enabled urban geographers and spatial scientist to quantitatively classify neighborhoods based on arrangement, shape, and structure of the built environment [24,25].

## Methodology

### Study area

This study area, Akure, is located in the south-western part of Nigeria. It is a medium-sized capital city of Ondo State, and it lies in the tropics at E 5°04′42"–E 5°29′45"/N 7°26′43"–N 7°03′50". Akure City, being the regional capital of Ondo State since 1976 is one of the emerging prominent urban centers. The city has a long history dated back to pre-colonial era, making it a traditional city with attractive responsibilities, such as the main base for Benin's trade and western frontier of Benin, and notably emerged as the headquarters of Ondo Province in 1915. The city has witnessed rapid population growth in the last twenty years. According to earlier study of Emmanuel [14] and Akinbamijo and Fasakin [16], Akure has three distinct morphological structures (zones), which are the core, transition, and peripheral. Further details about Akure have been discussed [10,11,26,27].

### Data and method

The study broadly used very high-resolution satellite imagery and gathered primary data from field survey. According to the field survey, malaria indicator questionnaire was used to gather malaria prevalence on U5. Details on the sampling and method of primary data collection have been discussed [27]. With the aid of the very high-resolution imagery, first, this study modeled and classified the urban structure of Akure based on building morphological characteristics, such as building size, building orientation, and density using the Momepy tool (https://docs.momepy.org/en/stable/) in python, according to the works of Fleischmann and Taubenböck et al. [28,29]. This was also augmented with expert classification, historical maps, and visual classification (qualitative). Second, this study used analysis of variance to statistically determine the difference in the mean of U5 malaria among the selected settlement classes. In this study, we randomly selected two settlements from each residential zones from the list of zones in Akure, as shown in Table 1.

**Table 1:** List of settlements and zonation in Akure.

Settlement classification			
Informal	Medium-density	Planned	Peri-Urban
Arakale	Fanibi/Lafe	Ijapo	Igoba
Erekesan	Ijomu	Alagbaka	Shagari
Isolo	Oke-Aro Titun	Oba-Ile Phase One	Owode
Isikan	Oke-Ogba	Oke-Ijebu	Adofure
Eruoba	Oke Padi	Ala	Ijoka Road
Odo Ikoyi	Aiyedun		Aule Road
Oritagun	Oke Isinkan		Gaga Area
Erekefa	Oke Arata		Ologede Area
Immagun			
Igann			

## Results

**Figure 2:**
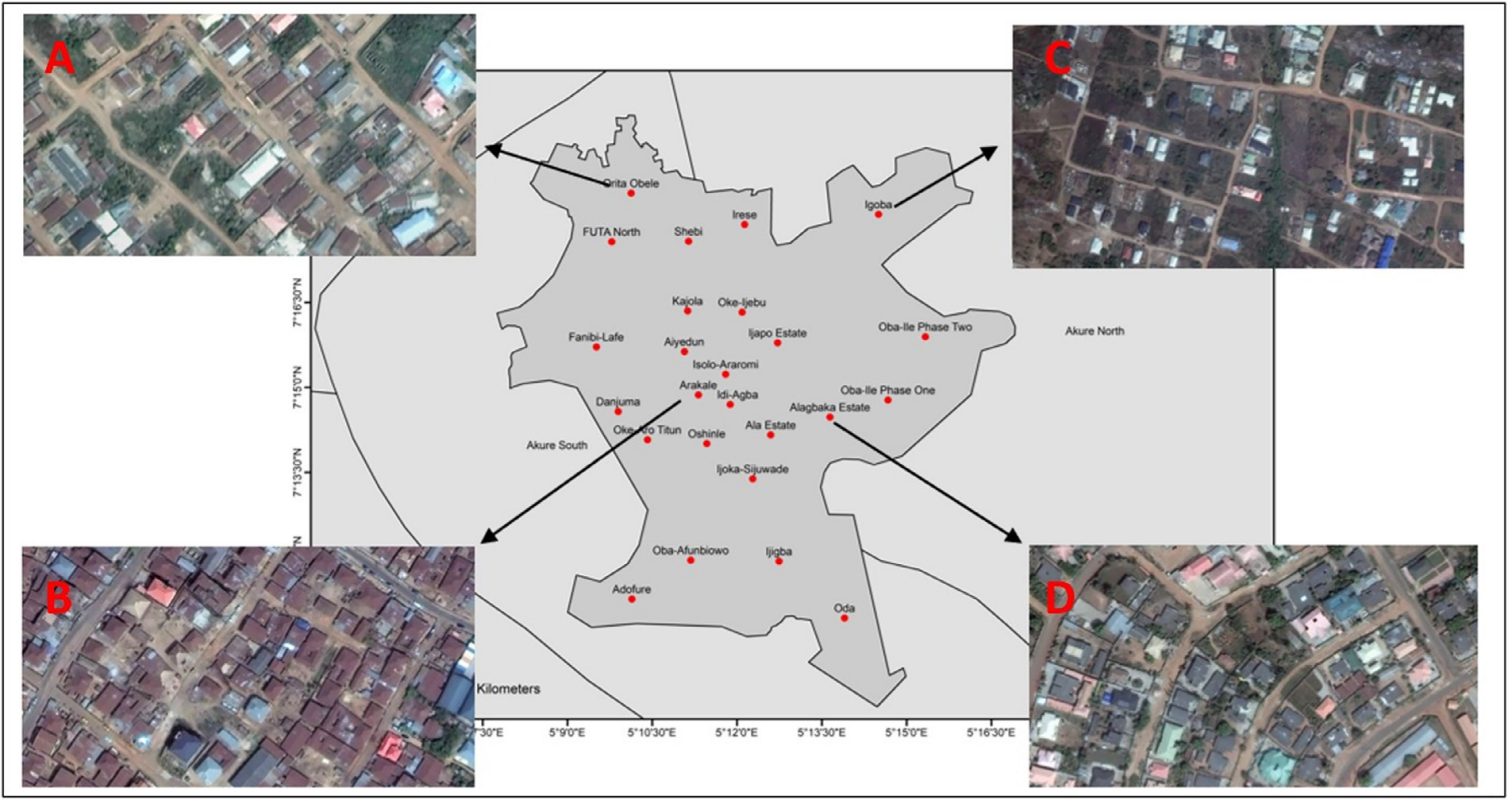
Study area with selected settlements.

**Figure 3:**
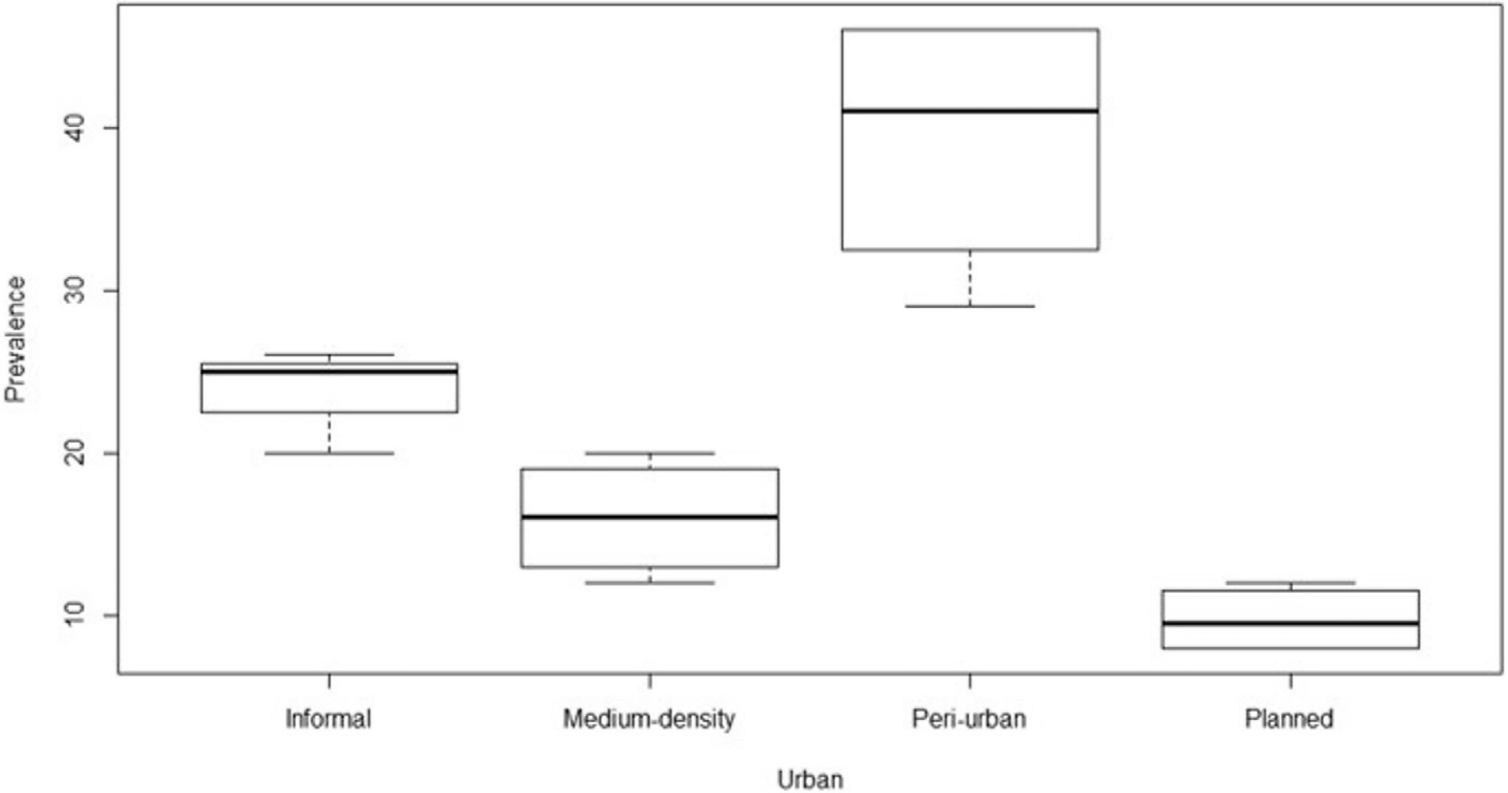
Analysis of variance result for the study.

The objectives of this study are to classify Akure into different morphological characteristics and to determine if disease burden (U5 malaria) is significantly different among the classified settlements. Based on the morphological characteristics, the study classified the settlements into four, which are medium-density, informal, peri-urban, and planned settlement, represented by letters A, B, C, and D, respectively, as shown in Figure 2. In a bid to test the significance difference of U5 malaria burden among the selected settlements, the analysis of variance result (Figure 3), according to the study analysis, shows that U5 malaria varies significantly among the settlement classes (*P* = 1.118e-05), with highest difference between peri-urban settlement and medium-density settlement (*P* = 0.0000988). There is no difference between planned and medium-density settlement and informal and medium settlement (*P* = 0.3091361 and *P* = 0.1440773, respectively). The study further shows that the highest burden of U5 malaria is associated with the peri-urban settlement. These areas are characterized with fragmented landscapes and weak urban planning [10,30].

## Discussion

The informal settlements are the core area or inner city. They represent the Central Business District, where the market, *Obas* palace, and earliest residential structures are located. These places lack formal or modern form of urban planning. Owoeye and Omole [31] argued that Akure follows Burgess’ concentric zone theory, where medium-density developments bridges informal and formal settlements. Our study supported this with distinct density characteristics between these settlements. The medium-density settlements are often occupied by middle-class residents [15,31]. The medium-density zone is followed by government residential estates earmarked for the affluent residents. This zone is also regarded as the high-class residential areas [31,32]. However, according to our study, we observed a distinction between the high-class residential areas (D) and characteristics of newly emerging areas (C), as shown in Figure 2. The newly emerging areas are the peri-urban settlements found at the outskirts of the city.

The core/informal and peri-urban settlements have higher burden of malaria than the medium-density and planned settlements. This is because much of urban expansion in Africa is characterized by unplanned and unregulated growth, exacerbated by the legacy of colonialism, structural adjustment, and neo liberalism that spawned weak urban planning institutions [33]. The unequal distribution and pattern of infrastructure are fallouts of weak planning controls in Akure. Infrastructures improve livability in communities and urban experiences. However, the study of Akinbamijo and Aladetuyi [34] on the state of infrastructure in Akure reveals that road, water, and health infrastructures are subpar. With focus on spatial distribution of health facilities in Akure, Oyinloye and Olugbamila [35,36] highlighted the inadequate distribution of health facilities among localities at the central wards. Furthermore, Olugbamila [36] echoed the need for town planners to ensure equitable distribution of health facilities in Akure while putting into consideration the location of existing functional health infrastructures. This is vital because since the birth of Akure, its development has been guided by an obsolete master plan, which was produced in 1980 [10].

## Recommendations and conclusion

The last two decades have witnessed enormous revolution in application of spatial science tools to city management, particularly, due to improved software computational capabilities, hardware, the fine scale resolution, and ubiquity of earth observation (EO) data. Despite these many advantages, cities in low- and middle-income countries lag in the study and modeling of the city spatial form. In this article, we modeled the urban residential structure of Akure using geographic information system technology and EO data sets. Furthermore, the study investigated the variability of malaria burden as a proxy for health outcome among the identified residential zones. The study identified variability in health outcomes, implying the indirect impact of urban structure on health outcome in Akure.

Paucity of data, such as EO, health, and location of health infrastructure, is a challenge and has limited the analytics of the study. However, this study highlights previous works done in Akure in this regard. We, therefore, strongly recommend extensive research with inclusion of the aforementioned data in their analysis.

## Declarations of competing interest

The authors have no competing interests to declare.
